# GP or ChatGPT? Ability of large language models (LLMs) to support general practitioners when prescribing antibiotics

**DOI:** 10.1093/jac/dkaf077

**Published:** 2025-03-13

**Authors:** Oanh Ngoc Nguyen, Doaa Amin, James Bennett, Øystein Hetlevik, Sara Malik, Andrew Tout, Heike Vornhagen, Akke Vellinga

**Affiliations:** CARA Network, School of Public Health, Physiotherapy and Sports Science, University College Dublin, Dublin, Ireland; CARA Network, School of Public Health, Physiotherapy and Sports Science, University College Dublin, Dublin, Ireland; NIHR In Practice Fellow, Hull York Medical School, University of Hull, Hull HU6 7RX, UK; Department of Global Public Health and Primary Care, University of Bergen, Bergen, Norway; Midleton Medi Center, Midleton, Co Cork, Ireland; Division of General Internal Medicine, Northwestern University Feinberg School of Medicine, Chicago, IL, USA; CARA Network, Insight Centre for Data Analytics, University of Galway, Galway, Ireland; CARA Network, School of Public Health, Physiotherapy and Sports Science, University College Dublin, Dublin, Ireland

## Abstract

**Introduction:**

Large language models (LLMs) are becoming ubiquitous and widely implemented. LLMs could also be used for diagnosis and treatment. National antibiotic prescribing guidelines are customized and informed by local laboratory data on antimicrobial resistance.

**Methods:**

Based on 24 vignettes with information on type of infection, gender, age group and comorbidities, GPs and LLMs were prompted to provide a treatment. Four countries (Ireland, UK, USA and Norway) were included and a GP from each country and six LLMs (ChatGPT, Gemini, Copilot, Mistral AI, Claude and Llama 3.1) were provided with the vignettes, including their location (country). Responses were compared with the country’s national prescribing guidelines. In addition, limitations of LLMs such as hallucination, toxicity and data leakage were assessed.

**Results:**

GPs’ answers to the vignettes showed high accuracy in relation to diagnosis (96%–100%) and yes/no antibiotic prescribing (83%–92%). GPs referenced (100%) and prescribed (58%–92%) according to national guidelines, but dose/duration of treatment was less accurate (50%–75%). Overall, the GPs’ accuracy had a mean of 74%. LLMs scored high in relation to diagnosis (92%–100%), antibiotic prescribing (88%–100%) and the choice of antibiotic (59%–100%) but correct referencing often failed (38%–96%), in particular for the Norwegian guidelines (0%–13%). Data leakage was shown to be an issue as personal information was repeated in the models’ responses to the vignettes.

**Conclusions:**

LLMs may be safe to guide antibiotic prescribing in general practice. However, to interpret vignettes, apply national guidelines and prescribe the right dose and duration, GPs remain best placed.

## Introduction

Inappropriate prescribing of antibiotics is a major cause of antimicrobial resistance, also referred to as the silent pandemic, as it has the potential to render many infections resistant to common antibiotics.^1^ Up to 80% of antibiotics are prescribed in general practice and many interventions aim to address inappropriate prescribing.^2^ Antibiotic prescribing in European general practice also shows wide variability in antibiotic prescribing practices. When evaluating antibiotic prescribing by indication and according to national guidelines, inconsistencies were observed in the adherence to recommended guidelines, with frequent overprescribing of broad-spectrum antibiotics or prescribing of antibiotics when they are not indicated.^3^ Similarly, research in the UK highlighted considerable variability in antibiotic prescribing across general practices, with changes in prescribing patterns not consistently aligning with updates to national guidelines.^4^

Large language models (LLMs) are artificial intelligence (AI) systems trained on extensive textual data such as books, articles and online content, with human-like capabilities such as providing summaries, translations and answers to questions. In 2018, the first LLM, generative pre-trained transformer 1 (GPT-1), was released by Open AI.^5^ This has been followed by the release of many other LLMs by big tech companies, such as Meta’s Llama and Google’s Gemini.^6,7^ LLMs can be improved through ‘training’ the models by providing questions and answers or other (updated) specific information.^8^ LLMs have been increasingly employed to assist clinical diagnosis, clinical decision support, virtual medical assistants and health chatbots, as well as automated medical report synthesis.^9^ LLMs have performed well on medical examinations and the selection of antidepressants, as well as supporting clinical decision-making and identification of drug–drug interactions^10–13^

A recent study showed the applicability of LLMs to support treatment suggestions for patients with resistant infections.^14,15^ A correspondence to the *Lancet Infectious Diseases*, with one of the nine US-based scenarios not solely based within the hospital, presented overall good responses from ChatGPT but also identified deficits in situational awareness, inference and consistency.^16^ No study based in general practice was identified. This study aimed to understand if LLMs provide accurate advice on antibiotic treatment and patient management in a general practice setting.

## Methods

Twenty-four vignettes were selected from the literature, which included upper respiratory tract infection, pneumonia, bronchitis, pharyngitis, urinary tract infection (UTI), COPD Gold II and III exacerbation, otitis media, cellulitis, sinusitis, acne, asymptomatic bacteriuria (ASB), wound, sore throat, varicella and dental infections.^17–24^ The decision to prescribe an antibiotic was guided by the description given in the vignette. Four countries were identified: USA, UK and Ireland, each with English and online prescribing guidelines, and Norway, as a different language option but with easy-to-find and accessible online guidelines. For each vignette, a country-specific treatment plan (antibiotic yes/no, which one, duration, other information) was based on the national guidelines for antibiotic prescribing: IDSA; NICE prescribing guidelines; the Irish antibiotic prescribing guidelines; and the Norwegian antibiotics prescribing guidelines in primary care.^25–28^ Based on the vignettes, 12 out of 24 advised to prescribe an antibiotic.

In each country, a practising GP, without a specific interest in antibiotic prescribing, was approached and asked to look at each vignette and provide a treatment plan as they would in daily clinical practice.

Six LLMs [ChatGPT (GPT-4o), Gemini, Copilot, Mistral Large 2, Claude 3.5 Sonnet and Llama 3.1 (8 billion parameter model)] were used (Table 1). ChatGPT is a chatbot, based on OpenAI’s most recent generative pre-trained transformer and known for its advanced capabilities for smoother human–computer interaction and generation of output in a very short time.^29^ Google’s Gemini, previously known as Bard, is designed to integrate with Google’s infrastructure but may be less flexible.^7^ Mistral AI is designed to handle very complex tasks and more specialized use within smaller contexts but may not perform as well with more creative tasks. Anthropic’s Claude is able to capture sophisticated instructions, write content with high quality and is focused on safer and more understandable AI outputs.^30,31^ Llama 3.1 is Meta’s LLM and works best within this environment. Llama is focused on flexibility and accessibility and has three versions: 8 billion (used in this study); 70 billion; and 405 billion.^32^

**Table 1.** Overview of features of the six LLMs used

Feature	ChatGPT (GPT4o)	Gemini	Copilot	Mistral Large 2	Claude 3.5 Sonnet	Llama 3.1
Developer	OpenAI	Google DeepMind	Microsoft	Mistral AI	Anthropic	Meta
Country of origin	USA	USA	USA	France	USA	USA
Training data Sources	Web pages, books, articles and licensed datasets	Web pages, books, scientific papers and proprietary data	Code repositories (e.g. GitHub), documentation and open-source projects	Web pages, books and multilingual datasets	Web pages, books and curated datasets for ethical alignment	Web pages, books and open-source datasets
Primary use cases	General-purpose AI, chatbots, coding, Q&A	Multimodal tasks, research, creativity	Code generation, developer assistance	General-purpose AI, multilingual tasks	General-purpose AI, ethical AI, Q&A	Research, open-source applications
Strengths	High accuracy, large context window	Multimodal capabilities, strong reasoning	Excellent for coding, integrates with IDEs	Efficient, multilingual, lightweight	Ethical alignment, strong reasoning	Open-source, customizable, cost-effective
Open source	No	No	No	Partially	No	Yes
Performance	State-of-the-art for general tasks	Strong in multimodal and creative tasks	Best for coding and developer tasks	Efficient for multilingual tasks	Strong in ethical reasoning and Q&A	Good for research and customization

IDE, integrated development environment.

A standardized approach was taken to prompt the LLMs, mirroring the GPs but with inclusion of country. All questions were presented in English. The baseline comparison was the US guidelines (and the vignettes without country specification). Each LLM and GP was prompted with the same questions: diagnosis; yes/no antibiotic; if yes, antibiotic choice, dose and duration; any advice provided; and which guidelines were used (if any). The prompts were run on each LLM between 7 August and 16 August 2025, while the GPs provided their advice between 7 August and 10 August 2025.

The percentage of accurately provided prescribing/treatment options was assessed for the GPs and LLMs by comparing with the national guidelines.

Additional evaluations were for hallucination, which occurs when the LLM generates a response that appears to be correct, but is useless and incorrect, and toxicity, which is defined as the presence of disrespectful, rude, unreasonable or aggressive comments.^33,34^ Hallucination was evaluated with the BERTScore, which evaluates the responses from LLMs to their prompts for relevance and out-of-context answers. Data leakage, which in this context can be defined as the ‘leakage of user’s input data’, was checked with Python (‘detect entities’ in Spacey library).^35^

## Results

A total of seven questions (diagnosis, antibiotic yes/no, choice of antibiotics, dose and duration, advice to patient, reference yes/no, which reference) prompted to six LLMs and four GPs were included.

**Figure 1.**
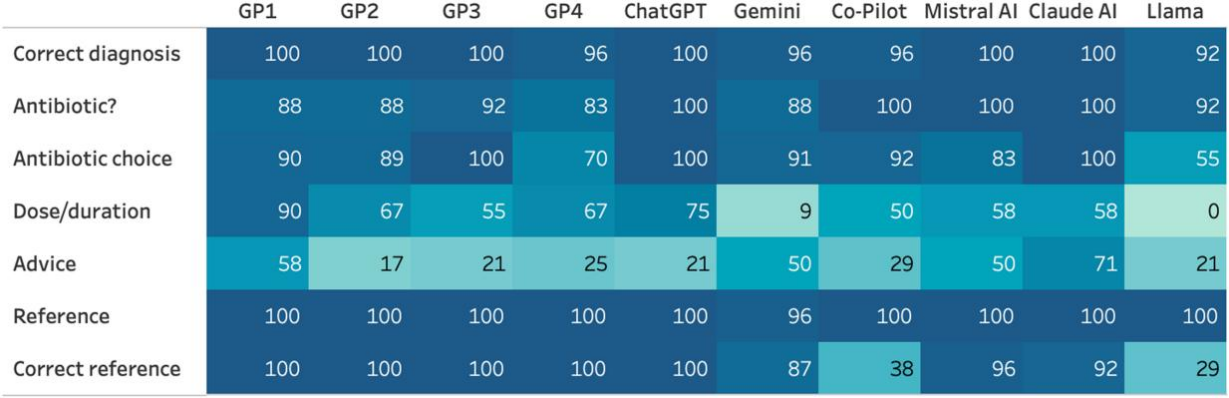
Heatmap presenting differences between GPs and LLMs in response to vignettes. Darker colour represents higher accuracy.

When considering the GPs’ answers to the vignettes, their accuracy in relation to diagnosis (96%–100%) and yes/no antibiotic prescribing (83%–92%) was high (Table 2 and Figure 1). Once this decision to prescribe or not was made, between 70% and 100% identified the right antibiotic according to their national guidelines, with the dose/duration provided being less accurate (55%–90%). All GPs were aware of their country’s antibiotic guidelines and could provide the right link for this. Advice to patients was not provided or was not added to the vignettes in 17%–58%. The overall mean score for the GPs was 79%.

**Table 2.** Overview of percentage correct answers for the 24 vignettes by GP and country

					USA					
	GP 1	GP 2	GP 3	GP 4	ChatGPT	Gemini	Copilot	Mistral AI	Claude	Llama
Correct diagnosis	100	100	100	96	100	96	96	100	100	92
Antibiotic?	88	88	92	83	100	88	100	100	100	92
Antibiotic choice	75	67	92	58	100	83	92	83	100	50
Dose/duration	75	50	50	50	75	8	50	58	50	0
Advice	58	0	21	25	21	50	29	50	71	21
Reference	100	100	100	100	100	96	100	100	100	100
Correct reference	100	100	100	100	100	83	38	96	92	29
Overall mean	83	67	76	69	83	68	68	81	85	49

GPs were compared with their own country’s guidelines.

In comparison with the GPs, the LLMs’ answers to the standard vignettes showed that each LLM scored highly in relation to diagnosis (92%–100%), antibiotic prescribing (88%–100%) and the choice of antibiotic (55%–100%), which showed similar variation as the GPs. However, the dose and duration provided by the LLMs varied from 0% to 75%. Between 21% and 71% of the LLMs provided advice; the best advice was provided by Claude (71%). References were provided, as asked in the prompts; however, the appropriateness of the references varied widely, from 29% to 100%.

**Figure 2.**
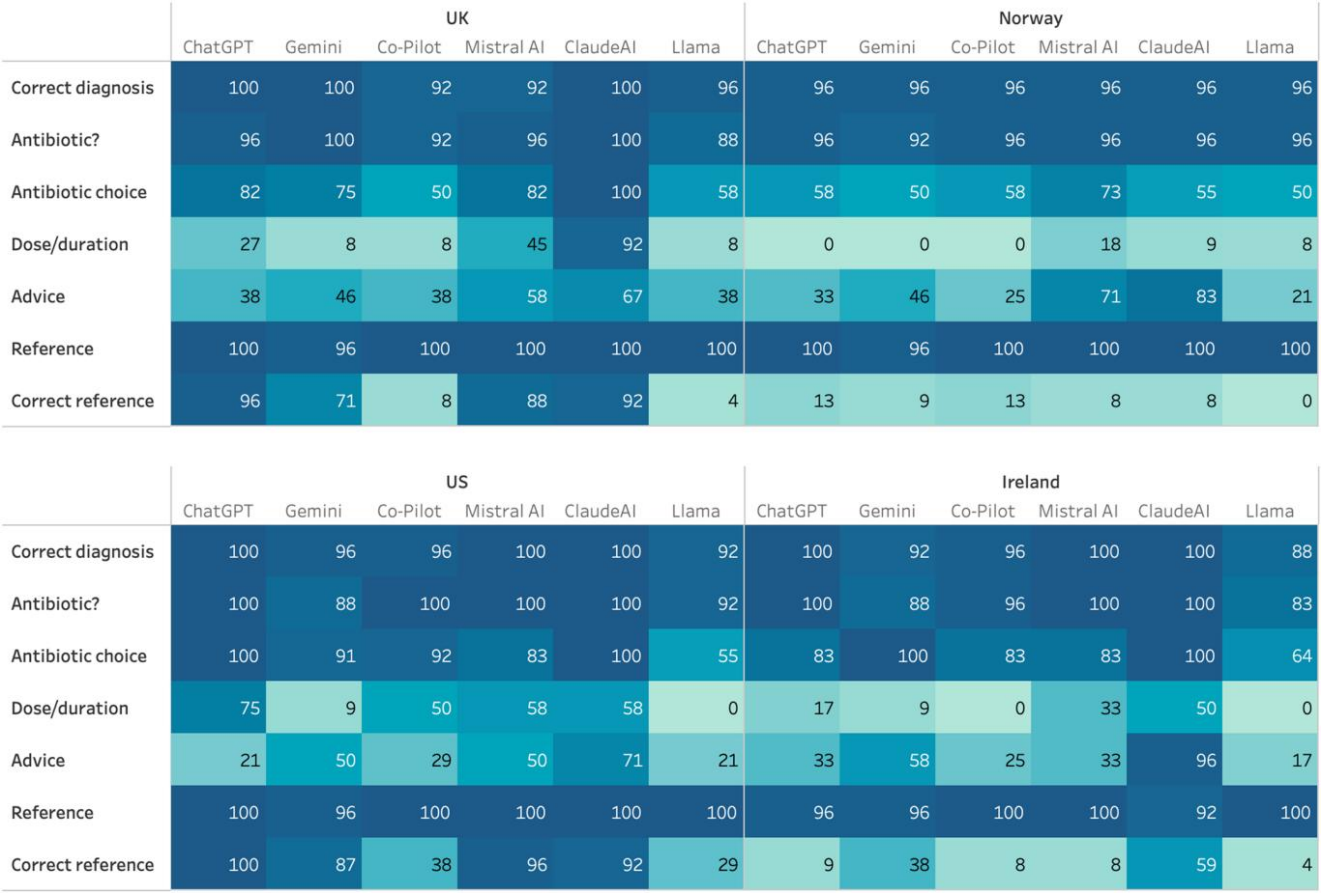
Overview of accuracy of LLMs in response to vignettes—country comparison.

Comparison of the LLMs using the country-specific vignettes, showed that for the standard (USA) the highest scores were observed for Claude (87%). ChatGPT scored similarly highly (83%), except for providing advice (21%). ChatGPT was the only model able to retrieve all the correct US references for its answers, followed by Mistral AI (96%) and Claude (92%). When comparing between the countries (Table 3 and Figure 2), diagnosis, antibiotic decision and choice were generally well provided; however, the application of the national guidelines, considering dose and duration, providing advice and identifying the appropriate reference were poor. The UK guidelines were better referenced than the Irish guidelines, in particular for ChatGPT (96% versus 9%), Claude (92% versus 59%) and Gemini (71% versus 38%). However, for the Norwegian guidelines, all LLMs performed very poorly (0%–13%). The overall mean of the LLMs, considering all vignettes and countries, was highest for Claude (80%), followed by Mistral AI (70%), ChatGPT (65%) and Gemini, Copilot and Llama (62%, 55% and 47%, respectively).

**Table 3.** Overview of percentage correct answers for the 24 vignettes, prompted with country, by each LLM compared with the specific guidelines of each country: Ireland, UK and Norway

	Ireland						UK						Norway					
	ChatGPT	Gemini	Copilot	Mistral AI	Claude	Llama	ChatGPT	Gemini	Copilot	Mistral AI	Claude	Llama	ChatGPT	Gemini	Copilot	Mistral AI	Claude	Llama
Correct diagnosis	100	92	96	100	100	88	100	100	92	92	100	96	96	96	96	96	96	96
Antibiotic?	100	88	96	100	100	83	96	100	92	96	100	88	96	92	96	96	96	96
Antibiotic choice	83	92	83	83	100	58	75	75	50	75	100	50	58	50	58	67	50	50
Dose/duration	17	8	0	33	50	0	25	8	8	42	92	8	0	0	0	17	8	8
Advice	33	58	25	33	96	17	38	46	38	58	67	38	33	46	25	71	83	21
Reference	96	96	100	100	92	100	100	96	100	100	100	100	100	96	100	100	100	100
Correct reference	8	38	8	8	54	4	96	71	8	88	92	4	13	8	13	8	8	0
Overall mean	56	63	52	60	82	44	72	66	49	76	92	48	50	49	49	60	58	46

The BERTScore (hallucination) ranged between 0.68 and 0.82, reflecting moderate semantic similarity and indicating that the models are not hallucinating. However, human expertise was still needed to determine whether the antibiotic treatment proposed was in line with the national prescribing guideline. No toxicity (threats, insults or attacks on identity) was observed in any of the responses obtained from the LLMs. However, data leakage (any leakage of personal information) was observed in all the responses of the vignettes that included a patient’s name or their initials.

## Discussion

Compared with GPs, LLMs evaluated against US guidelines, show high reliability in the decision to prescribe an antibiotic and the choice of antibiotic, as well as identifying the diagnosis described in the vignettes, whereas the dose/duration was less accurate. When comparing the performance of LLMs between countries, and against the country’s specific guidelines, LLMs often fail to provide accurate references to national guidelines and recommendations. GPs were reliable in diagnosis and the decision to prescribe, but importantly, more reliable in referencing and applying national guidelines.

The vignettes were written for antibiotic prescribing and for 12 out of 24 vignettes (50%) an antibiotic was indicated. For the vignettes where prescribing was indicated, accuracy in the choice of antibiotic was similar for LLMs and GPs, irrespective of the country, as the type of antibiotic would be relatively similar for different countries, due to their range and mechanism of action.^36^

The accuracy of LLMs reduces when more detailed (country-specific) information is required, which suggests that LLMs are largely trained on US information, as this was the most accurate model. However, information on the sources of data accessed by LLMs is not easily available, or has not been released.^37^

A strength of some LLMs was the inclusion of advice, in particular Claude, even though this has to be interpreted with caution. GPs may not have expanded on advice in their responses on the vignettes, which in daily clinical practice would be guided by the patient, and their questions. Also, the appropriateness of LLMs providing advice was challenged in a comparative vignette study with nurses, which showed that LLMs had a tendency towards over-triaging and too much information leading to indecisiveness.^12^ Furthermore, the importance of human interpretation was clear as the LLM answers had to be interpreted in line with the national guidelines and checked individually. Human expertise regarding the application of the national guidelines may rely on the nuance and interpretation of experienced GPs; however, the answers regarding advice to patients did not provide enough evidence about this point. Assessment of advice, together with follow-up of the outcome for patients or, alternatively, the simultaneous application of LLMs in real-life consultations would be a logical next step.

A wide variety was observed between different LLMs, reflecting the variety in the training data, as well as the contextual interpretation and model capabilities. More studies compare the quality of answers between LLMs, and between LLMs and human decision.^10,38^ Depending on the context, training data and model capacity, LLMs performed well, though often suffering from a lack of interpretation, incorrect statements and a lack of references. ChatGPT has been shown to recognize clinically important factors when explicit information was provided but missed relevant issues in scenarios of increasing complexity.^16^ In the presented comparison, however, the GPs, probably due to a more nuanced interpretation and inherent understanding of national guidelines, outperformed LLMs, except for Claude. LLMs can be improved in this area by more training data but also through algorithms that apply to country-specific guidelines and/or other factors such as the inclusion of different languages.

Of particular interest in relation to LLMs is the concerns in relation to data privacy and security, and a tailored approach to regulatory oversight has been suggested previously.^37,39^ From our findings, some leakage occurred, when patient information was included in the vignettes. This should be a real concern for GPs and a warning never to include patient details when using LLMs.^40^

In a recent, but not yet reviewed, paper on how LLMs can influence medical decision-making, clinicians were asked to assess triage, risk and treatment before and after receiving advice generated by ChatGPT.^41^ Clinicians were willing to change their decisions based on the AI assistance, and improvements could be made. Considering our results, in particular the lack of nuance and application of national guidelines, the antibiotic prescribing decision should remain with the GP; however, LLMs can be used to provide advice.

The study showed the potential of LLMs to suggest when antibiotic treatment is appropriate, potential antibiotic agents and advice. However, GPs or other prescribers of antibiotics should interpret and use the information with caution and awareness of its limitations. Whereas the results have shown that LLMs can support antibiotic prescribing, the identification and implementation of national guidelines is suboptimal and there are potential concerns in relation to patient privacy. Improvements can be made by optimizing model training and the application of relevant algorithms.
