# Correction to: GP or ChatGPT? Ability of large language models (LLMs) to support general practitioners when prescribing antibiotics

**DOI:** 10.1093/jac/dkaf237

**Published:** 2025-07-08

This is a correction to: Oanh Ngoc Nguyen, Doaa Amin, James Bennett, Øystein Hetlevik, Sara Malik, Andrew Tout, Heike Vornhagen, Akke Vellinga, GP or ChatGPT? Ability of large language models (LLMs) to support general practitioners when prescribing antibiotics, *Journal of Antimicrobial Chemotherapy*, Volume 80, Issue 5, May 2025, Pages 1324–1330, https://doi.org/10.1093/jac/dkaf077.

The originally published version of this manuscript omitted to designate Oanh Ngoc Nguyen and Doaa Amin as joint first authors. This error has been corrected.

